# CD11c regulates late-stage T cell development in the thymus

**DOI:** 10.3389/fimmu.2022.1040818

**Published:** 2022-11-10

**Authors:** Lifei Hou, Koichi Yuki

**Affiliations:** ^1^ Department of Anesthesiology, Critical Care and Pain Medicine, Cardiac Anesthesia Division, Boston Children’s Hospital, Boston, MA, United States; ^2^ Departments of Anaesthesia and Immunology, Harvard Medical School, Boston, MA, United States

**Keywords:** CD11c, thymus, dendritic cell, apoptosis, T cell development

## Abstract

CD11c, also named integrin αX, has been deemed solely as a dendritic cell marker for decades while the delineation of its biological function was limited. In the current study, we observed in mice that CD11c deficiency led to a defect in T cell development, demonstrated by the loss of CD4^+^CD8^+^ double positive (DP) T cells, CD4^+^CD8^-^, and CD4^-^CD8^+^ single positive (SP) T cells in the thymus and less mature T cells in the periphery. By using bone marrow chimera, we confirmed that CD11c regulated T cell development in the thymus. We further showed that CD11c deficiency led to an accelerated apoptosis of CD3 positive thymocytes, but not CD4^-^CD8^-^ double negative (DN) T cells. Overall, this study added one more layer of knowledge on the regulatory mechanism of late-stage T cell development that the presence of CD11c in the thymus is critical for maintaining T cell survival.

## Introduction

The thymus is the primary lymphoid organ that supports T cell development consisting of three main stages (double negative (DN), double positive (DP), and single positive) (1), during which a dynamic relocation of developing lymphocytes within multiple architectural structures occurs (2). During the last two decades, it has been well elucidated that two crucial decision steps, positive and negative selections, are needed to produce functional major histocompatibility complex (MHC)-restricted T cells, while simultaneously restricting the production of auto-reactive T cells (3, 4). The traditional knowledge is that cortical thymic epithelial cells (cTECs) are involved in thymocyte positive selection, and medullary thymic epithelial cells (mTECs) and dendritic cells (DCs) are involved in negative selection (5, 6). While it is well known that events, such as T cell receptor (TCR) *β* chain rearrangement (7, 8), proper TCR-MHC affinity and signaling strengths (9–12), finely regulate positive and negative selections, the regulation of late-stage T cell maturation, survival, and emigration in the thymus is less studied (5).

β2 integrins are called leukocyte integrins, exclusively expressed on leukocytes (13–15). They consist of four members CD11a/CD18 (αLβ2), CD11b/CD18 (αMβ2), CD11c/CD18 (αXβ2), and CD11d/CD18 (αDβ2) (16). CD11c has been deemed primarily as a dendritic cell (DC) marker (17–19), and its physiological function hasn’t been extensively explored. Our recent study revisited CD11c and discovered that it is also expressed on hematopoietic stem and progenitor cells (HSPCs), and its deficiency leads to the loss of HSPCs through an enhanced apoptosis in sepsis and bone marrow transplantation mouse models (20). In the study, we reported that CD11c (αX) knockout (KO) mice showed lower CD3 T cell counts in peripheral blood. Motivated by this clue, we further explored the biological function of CD11c, and discovered that it played a pivotal role in maintaining T cell survival at the late-stage development in the thymus.

## Results

**Figure 1:**
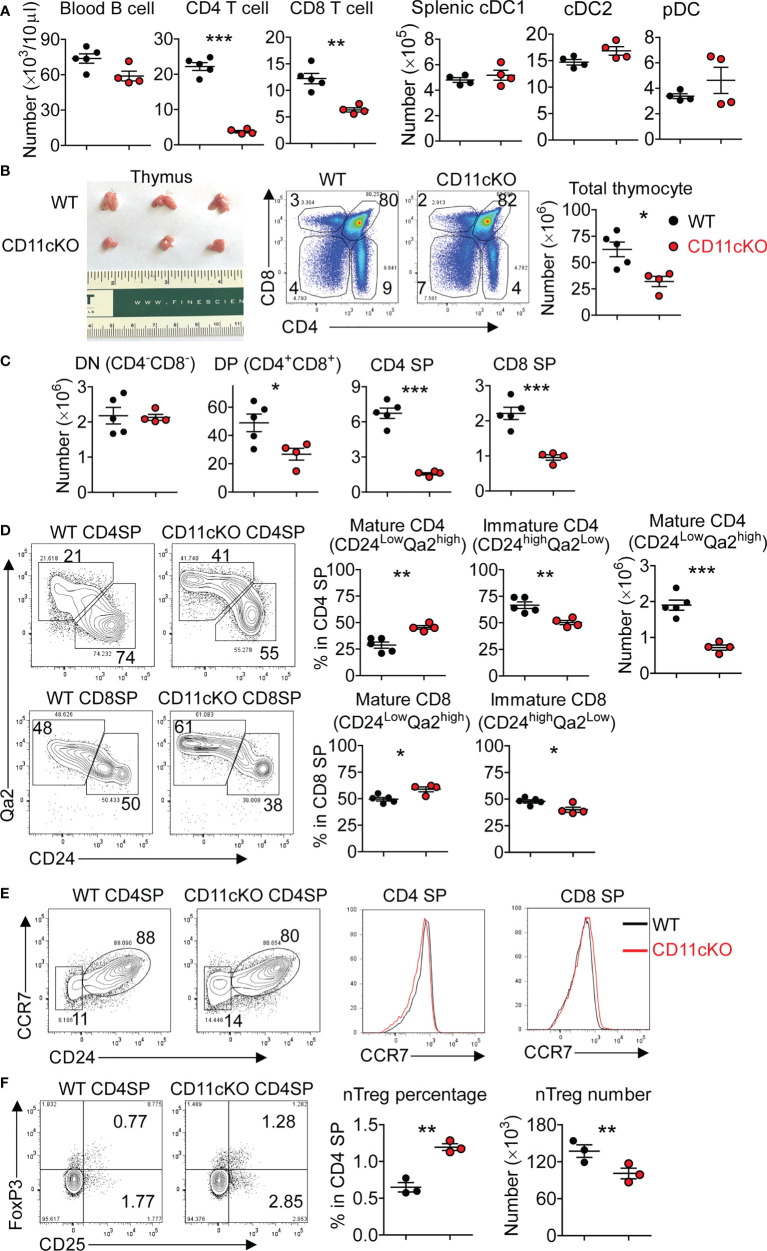
Thymic atrophy in CD11c KO mice. **(A)** Left: T and B cell counting in the peripheral blood in naïve WT and CD11c KO mice; Right: dendritic cell subset counting in the spleen. cDC1 was gated as MHC-II^+^XCR1^+^CD8a^+^, cDC2 as MHC-II^+^XCR1^-^CD8a^-^SIRPα^+^CD11b^+^, and pDC as PDCA^+^CD11b^-^Ly6C^+^. **(B)** Left: thymus image; Middle: representative FACS data, gated on total thymocytes of WT and CD11c KO mice; Right: dot plots of thymocyte number. **(C)** Thymic T cell subset numbers. DN was gated as CD4^-^CD8^-^, DP as CD4^+^CD8^+^, CD4 SP as CD4^+^CD8^-^, and CD8 SP as CD4^-^CD8^+^. **(D)** Left: representative FACS data showing maturation status of thymic CD4 SP and CD8 SP cells of WT and CD11c KO mice; Middle: dot plot showing percentage of immature and mature subsets in total CD4 and CD8 SP cells; Right: dot plot showing absolute number of mature CD4 SP cells; **(E)** Representative FACS data showing CCR7 expression on thymic CD4 SP and CD8 SP cells of WT and CD11c KO mice; **(F)** Left: representative FACS data showing natural regulatory T cells (nTreg) in thymic CD4 SP cells of WT and CD11c KO mice; Right: dot plots of both percentage and absolute number. nTreg cells were gated as CD25^+^FoxP3^+^CD4^+^ cells. Experiments were repeated at least 2-3 times with the same pattern. Student t test was performed for statistical analysis. *p < 0.05, **p < 0.01, ***p < 0.001.

We compared the peripheral blood leukocytes in naïve wild type (WT thereafter) and CD11c KO mice, and found that, even at steady status, CD11c deficiency led to a significant loss of CD4 and CD8 T cells (**Figure 1A**), which was also the case in the spleen (data not shown). Since CD11c is a marker of DCs, on which MHC-II molecules are expressed to critically maintain the number of peripheral T cells (21), we examined the number of DCs. Surprisingly, although CD11c KO mice had a relatively smaller size of spleen, the number of splenic DCs including conventional DC1 (cDC1, MHC-II^+^XCR1^+^CD8a^+^), cDC2 (MHC-II^+^XCR1^-^CD8a^-^SIRPα^+^CD11b^+^), and plasmacytoid DC (pDC, PDCA^+^CD11b^-^Ly6C^+^) subsets was not different from that of WT mice (**Supplemental Figure 1**, **Figure 1A**), suggesting that CD11c deficiency didn’t abrogate DC development *in vivo*. We then compared the thymus, the central lymph organ for T cell development. Surprisingly, for the first time, we showed the T cell development was defective in CD11c KO mice, manifested by the smaller-sized thymus with the loss of cellularity (**Figure 1B**). We performed detailed phenotyping of thymic T cells at different developmental stages, which revealed that CD11c deficiency was associated with the loss of DP, CD4 SP and CD8 SP cells, but exerted no influence on the number of DN cells (**Figure 1C**). Further analysis showed that, although less SP CD4 and CD8 cells existed in the thymus of CD11c KO mice, they were skewed toward more mature population, demonstrated by a higher ratio of CD24^low^Qa2^high^ cells (**Figure 1D**). This result indicated that immature CD4 SP and CD8 SP cells were particularly affected in the thymus of CD11c KO mice. Despite that the ratio of mature population in total CD4 SP was relatively higher, the absolute number of mature CD4 cells in thymus of CD11c KO mice was still significantly less than their WT counterpart (**Figure 1D**). CCR7 drives T cells from the cortex to the medulla (22, 23). CCR7 expression on CD4 and CD8 SP cells was not different between the genotypes, suggesting that the egress of T cells from the cortex to the medulla was comparable between WT and CD11c KO mice (**Figure 1E**). Strong TCR signal leads to negative selection, and weak signal helps to generate conventional CD4 cells (24, 25). Intermediate signal generates nTreg cells (26, 27). There was a relatively higher percentage of nTreg (CD25^+^FoxP3^+^CD4) cells in CD4 SP cells in the thymus of CD11c KO mice. This may indicate that nTreg pathway was less affected in CD11c KO mice compared to conventional CD4 pathway. Due to the lower number of total CD4 SP cells, however, the absolute number of nTreg in the thymus of CD11c KO mice was still less than the counterpart in WT mice (**Figure 1F**).

**Figure 2:**
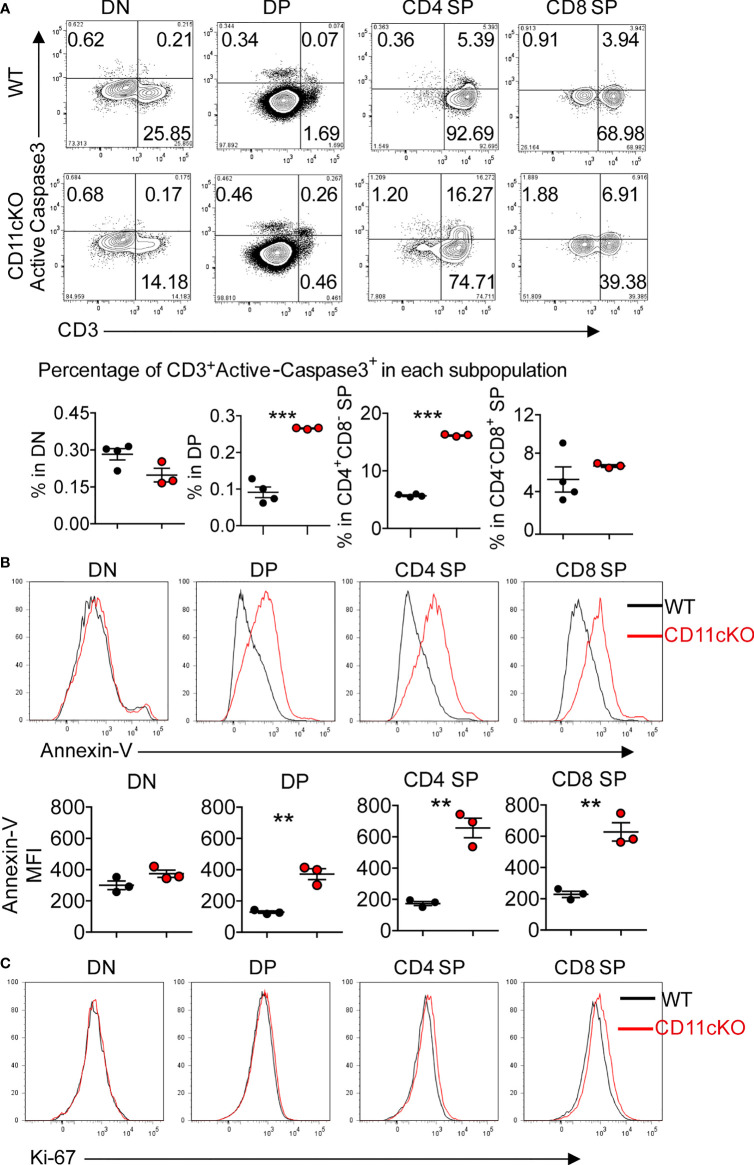
Apoptosis and proliferation analysis in CD11cKO mice. **(A)** Up panel: representative FACS data gated on different thymic T cell subsets with active cleaved caspase 3 expression; Bottom panel: Dot plots of percentage of CD3^+^Active-caspase3^+^ cells in indicated subsets. **(B)** Up panel: representative Annexin-V staining overlay analysis, gated on different thymic T cell subsets; Bottom panel: MFI. **(C)** Representative Ki-67 staining overlay analysis, gated on different thymic T cell subsets. Each symbol represents an individual mouse. Experiments were repeated at least 3 times with the same pattern. **(A)** and **(B)**, Student t test was used for statistical analysis. **p < 0.01, ***p < 0.001.

To explore the underlying mechanism that led to less thymocyte number in CD11c KO mice, we examined the apoptosis of T cells in the thymus by staining cleaved caspase-3 *ex vivo*. We found that CD11c deficiency significantly increased the apoptosis of CD3-positive subsets (DP, CD4 SP and CD8 SP cells), which are relatively more matured T cells in the thymus (**Figure 2A**). The more occurrence of apoptosis in CD3 positive cells was further confirmed by staining freshly isolated thymocytes with Annexin V (**Figure 2B**). In sharp contrast, the proliferation status was not different between the genotypes, probed by Ki-67 expression (**Figure 2C**).

**Figure 3:**
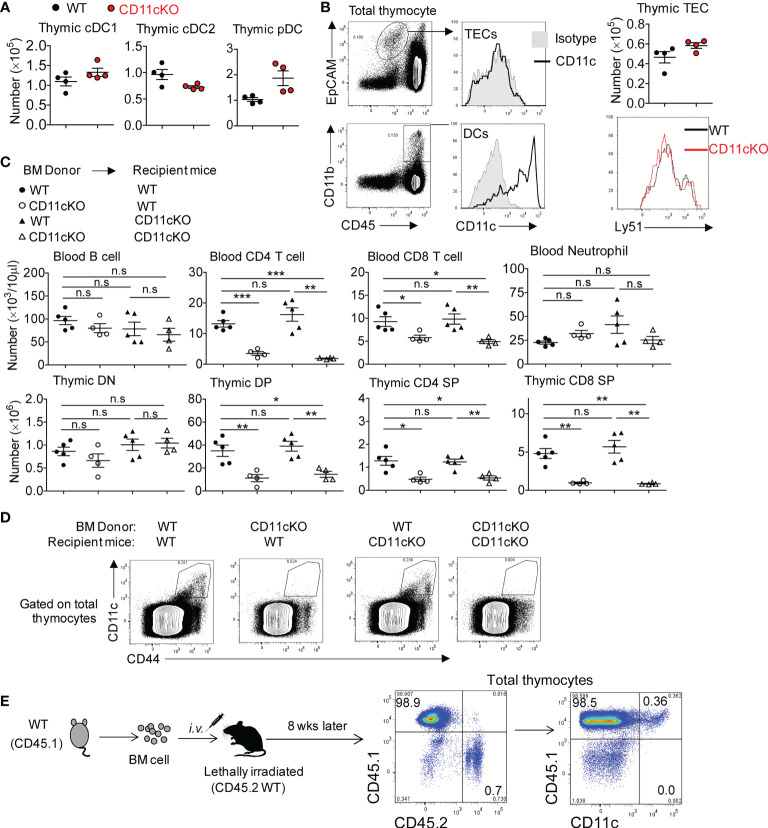
The role of CD11c in irradiation-sensitive hematopoietic cells in thymus. **(A)** thymic DC subset cell counting. cDC1 was gated as MHC-II^+^XCR1^+^CD8a^+^, cDC2 as MHC-II^+^XCR1^-^CD8a^-^SIRPα^+^CD11b^+^, and pDC as PDCA^+^CD11b^-^Ly6C^+^. Gating strategy could be found in **Supplement Figure 1**. **(B)** Left panel: representative FACS data showing CD11c expression pattern; Right-up panel: thymic TECs counting; Right- bottom panel: Ly-51 staining overlay analysis of TECs. **(C)** Blood (up panel) and thymic (bottom panel) analysis of bone marrow chimeric mice. **(D)** Representative FACS data showing CD11c expression pattern in thymocytes of chimeric mice described in **(C)**. **(E)** Representative FACS data showing CD11c expression pattern in thymocytes of chimeric mice by transferring CD45.1 WT bone marrow cells into lethally irradiated CD45.2 WT recipient mice. Each symbol represents an individual mouse. Experiments were repeated at least 3 times with the same pattern. **(A, B)**, Student t test was used for statistical analysis; **(C)** One-way ANOVA with Bonferroni *post hoc* analysis was performed. *p < 0.05, **p < 0.01, ***p < 0.001. n.s., no significant difference.

Successful TCR*β* chain rearrangement delivers proliferation signals and instructs the transition of DN cells into DP cells. This event is followed by positive selection by thymic epithelial cells (TECs) in the cortex and negative selection by DCs in the medullary region, responding to strong TCR-MHC interactions (28). Those DP cells with non-functional TCR-MHC interactions undergo death by neglect, which occurs for over 95% of DPs (29). To dissect out the cell type primarily responsible for the observed phenotype, we compared thymic DC subsets between WT and CD11c KO mice and found the number of three DC subset was comparable (**Figure 3A**). Although TECs are important antigen presenting cells in the thymus, no CD11c expression was detected on the surface of TECs (**Figure 3B**). In addition, the number of TECs between WT and CD11c KO mice was not different (**Figure 3B**). TECs contain two subpopulations; Cortical thymic epithelial cells (cTECs, Ly51^+^), which are the primary cell type involved in thymocyte positive selection, and medullary thymic epithelial cells (mTECs), which are involved in negative selection. We compared these two subpopulations by probing Ly51 expression and didn’t observe the difference between the genotypes (**Figure 3B**). Thus, TECs were excluded from the potential contributor to the phenotype observed in CD11c KO mice. CD11c was highly expressed on DCs, as expected (**Figure 3B**). CD11c was not detected on DN, DP, and SP T cells (data not shown). To further verify whether DCs in the thymus were responsible for the T cell maturation defect in CD11c KO mice, we created bone marrow (BM) chimera. Recipient mice, either lethally irradiated WT or CD11c KO mice were transplanted with either WT-derived or CD11c KO-derived bone marrow cells. Six weeks after the transplantation, peripheral blood leukocytes were monitored to ensure the success reconstitution of hematopoietic system. Mice were sacrificed at 8 weeks post BM transplantation. As shown in **Figure 3C**, as long as the donor BM cells were derived from CD11c KO mice, T cell development defect was observed regardless of the background of recipient mice, consistent with what we observed in CD11c KO mice. CD11c expression was done in the thymus of chimeric mice (**Figure 3D**). To further solidify our finding that CD11c-expressing cells in the thymus is irradiation sensitive and also to exclude the error due to mouse background, we also made the chimera mice in an opposite way. We used CD45.1 WT as donor and CD45.2 WT as recipient mice. We confirmed that all CD11c positive cells are donor (CD45.1) derived ones (**Figure 3E**). Thus, combined with the result of CD11c expression analysis in the thymus of chimeric mice (**Figures 3D, E**), we concluded that CD11c in irradiation-sensitive hematopoietic cells unexpectedly played an essential role in maintaining T cell survival in the thymus.

## Discussion

The current study discovered that CD11c was essential in regulating thymic T cell development by maintaining the survival of T cells at later stages of the development, which adds additional nodes to both T cell biology and DC function in the thymus. While positive and negative selections are well studied, late-stage T cell maturation in the thymus and its emigration into the periphery are less examined. Herein, we discovered that CD11c was critical in maintaining the survival of T cells, preferably CD3-positive ones, thus adding one more layer of knowledge on the regulatory mechanism of T cell maturation in the thymus.

Regarding the role of DCs in thymic T cell development, controversial reports have been made. On one side, thymic DCs have been deemed as a major player that mediates negative selection to induce the apoptosis of DP cells (28, 30, 31); on the other side, thymic DCs were also reported to be involved in positive selection, thus to maintain the survival of DP cells (32, 33). The discovery that CD11c plays an essential role in maintaining the survival of T cells suggests that, in addition to MHC-II molecule, DCs could use CD11c to maintain T cell survival. Interestingly, we found that CD24^hi^ immature CD4 T cells were selectively depleted in CD11c KO mice. CD24^hi^ immature CD4 SP cells are defined as “semi-mature” and susceptible to apoptosis when triggered through TCR (1, 34). Thus, our data is in line with the previous reports describing that CD24^hi^ immature CD4 SP cells are more sensitive to apoptosis over CD24^lo^ mature CD4 SP cells.

Overall, this study highlights the role of CD11c as a functional molecule to maintain the survival of T cells in the thymic late-stage T cell development.

## Methods

### Mice

Animal studies were approved by the Institutional Animal Care and Use Committee of Boston Children’s Hospital. Wild type mice on the C57BL/6J background were purchased from Jackson laboratory and acclimated in our animal facility before use. CD11c germline knockout mice (CD11cKO mice) on the C57BL/6J background were kindly given by Dr. Ballantyne (Baylor University), as described in our previous publication (20). For experiments, 7~10 week-old mice were used. Flow cytometry, and cell counting were performed as previously described (35). Regarding TEC and DC detection, the thymus was digested by type IV collagenase (0.5 mg/ml) and DNase I (50 unit/ml) in RPMI-1640 containing 5% FCS for 30 minutes at 37°C, followed by washing and resuspension.

### Chimera experiment

To generate single bone marrow chimeras, recipient mice on the C57BL/6 background were irradiated with two doses of 550 rad with 4-hour intervals. WT or CD11c KO derived bone marrow cells (total of 5 × 10^6^ cells) were injected into the tail vein of lethally irradiated recipients (WT or CD11c KO mice). Mice were evaluated for the reconstitution of the immune compartment after bone marrow transplantation. To prevent bacterial infection, the mice were provided with autoclaved drinking water containing sulfatrim for 1 week prior to and for 4 weeks after irradiation.

### Apoptosis analysis

Annexin-V staining method: Thymocytes were stained with fluorochrome conjugated antibodies to surface marker including CD3, CD4, CD8, and Annexin-V in the presence of Annexin-V binding buffer. After washing, cells were resuspended in Annexin-V binding buffer and collected freshly.

Active-caspase-3 method: Thymocytes were stained with fluorochrome conjugated antibodies to surface marker including CD3, CD4 and CD8. After washing, cells were fixed, permeabilized and stained intracellularly with fluorochrome-conjugated anti-active caspase 3-by using fixation/permeabilization reagents and protocols from BD Bioscience. In certain situation, intracellular Ki-67 staining was done together with active caspase-3 staining.

### Antibodies

Fluorochrome-conjugated antibodies or cell death related dyes are: from Biolegend: FITC- or PE-Cy7-anti-mCD3 (145-2C11), Pacific blue- or PE-Cy7- anti-mCD45.1 (A20), Pacific blue- or FITC- anti-mCD45.2 (104), Pacific blue- or PE-anti-mCD45 (30-F11), Pacific blue- or PE-Cy7- or APC- anti-mCD4 (GK1.5), APC-Cy7-anti-mCD8 (53-6.7), PE-Cy7-anti-mCCR7 (4B12), PE-Cy7-anti-mCD11b (M1/70), FITC-anti-mLy6C (HK1.4), Pacific blue- anti-mI-A/I-E (M5/114.15.2), APC-anti-mQa2 (695H1-9-9), APC-anti-mLy51 (6C3), PE-anti-mCD326 (Ep-CAM, clone G8.8), PE-anti-mCD25 (3C7), Alexa Fluor488-antiFoxP3 (FJK-16s). From eBioscience: PE-Cy7-anti-Ki67 (S01A15). From BD Biosciences: FITC-rabbit-anti-active caspase3 (C92-605), FITC-Annexin V, Annexin V staining buffer, and BD Cytofix/Cytoperm buffer. Cell counting was done by applying Sphero AccuCount beads (ACBP-50-10; Spherotech Inc, Lake Forest, IL). Data were acquired on a Canto II cytometer (BD Biosciences) and analyzed using FlowJo software (Tree Star).

### Statistical analysis

Statistical analyses were performed using Prism 4 (Graphpad Software). Student’s t-test, unpaired and paired, and one-way ANOVA were used according to the type of experiment. P value < 0.05 was considered significant.

## Data availability statement

The original contributions presented in the study are included in the article/**Supplementary Material**. Further inquiries can be directed to the corresponding authors.

## Ethics statement

The animal study was reviewed and approved by Institutional Animal Care and Use Committee of Boston Children’s Hospital.

## Author contributions

Both authors designed research, did experiment, analyzed data and wrote the manuscript. All authors contributed to the article and approved the submitted version.

## Conflict of interest

The authors declare that the research was conducted in the absence of any commercial or financial relationships that could be construed as a potential conflict of interest.

## Publisher’s note

All claims expressed in this article are solely those of the authors and do not necessarily represent those of their affiliated organizations, or those of the publisher, the editors and the reviewers. Any product that may be evaluated in this article, or claim that may be made by its manufacturer, is not guaranteed or endorsed by the publisher.

## References

[1] Kishimoto H Sprent J . Negative selection in the thymus includes semimature T cells. J Exp Med (1997) 185:263–71. doi: 10.1084/jem.185.2.263 PMC2196120 9016875

[2] Miller JF . The golden anniversary of the thymus. Nat Rev Immunol (2011) 11:489–95. doi: 10.1038/nri2993 21617694

[3] Robey E Fowlkes BJ . Selective events in T cell development. Annu Rev Immunol (1994) 12:675–705. doi: 10.1146/annurev.iy.12.040194.003331 8011294

[4] Takahama Y . Journey through the thymus: stromal guides for T-cell development and selection. Nat Rev Immunol (2006) 6:127–35. doi: 10.1038/nri1781 16491137

[5] Klein L Kyewski B Allen PM Hogquist KA . Positive and negative selection of the T cell repertoire: What thymocytes see (and don't see). Nat Rev Immunol (2014) 14:377–91. doi: 10.1038/nri3667 PMC4757912 24830344

[6] Takahama Y Ohigashi I Baik S Anderson G . Generation of diversity in thymic epithelial cells. Nat Rev Immunol (2017) 17:295–305. doi: 10.1038/nri.2017.12 28317923

[7] Mombaerts P Clarke AR Rudnicki MA Iacomini J Itohara S Lafaille JJ . Mutations in T-cell antigen receptor genes alpha and beta block thymocyte development at different stages. Nature (1992) 360:225–31. doi: 10.1038/360225a0 1359428

[8] Yuuki H Yoshikai Y Kishihara K Matsuzaki G Ayukawa K Nomoto K . The expression and sequences of T cell antigen receptor beta-chain genes in the thymus at an early stage after sublethal irradiation. J Immunol (1989) 142:3683–91. 2523931

[9] von Boehmer H . Developmental biology of T cells in T cell-receptor transgenic mice. Annu Rev Immunol (1990) 8:531–56. doi: 10.1146/annurev.iy.08.040190.002531 2188673

[10] Kisielow P von Boehmer H . Negative and positive selection of immature thymocytes: Timing and the role of the ligand for alpha beta T cell receptor. Semin Immunol (1990) 2:35–44. 2151796

[11] Gallegos AM Bevan MJ . Central tolerance: good but imperfect. Immunol Rev (2006) 209:290–6. doi: 10.1111/j.0105-2896.2006.00348.x 16448550

[12] Gascoigne NR Palmer E . Signaling in thymic selection. Curr Opin Immunol (2011) 23:207–12. doi: 10.1016/j.coi.2010.12.017 PMC3073816 21242076

[13] Springer TA Dustin ML . Integrin inside-out signaling and the immunological synapse. Curr Opin Cell Biol (2012) 24:107–15. doi: 10.1016/j.ceb.2011.10.004 PMC3294052 22129583

[14] Luo BH Springer TA . Integrin structures and conformational signaling. Curr Opin Cell Biol (2006) 18:579–86. doi: 10.1016/j.ceb.2006.08.005 PMC1618925 16904883

[15] Stewart M Thiel M Hogg N . Leukocyte integrins. Curr Opin Cell Biol (1995) 7:690–6. doi: 10.1016/0955-0674(95)80111-1 8573344

[16] Shimaoka M Springer TA . Therapeutic antagonists and conformational regulation of integrin function. Nat Rev Drug Discov (2003) 2:703–16. doi: 10.1038/nrd1174 12951577

[17] Steinman RM Pack M Inaba K . Dendritic cells in the T-cell areas of lymphoid organs. Immunol Rev (1997) 156:25–37. doi: 10.1111/j.1600-065X.1997.tb00956.x 9176697

[18] Corbi AL Garcia-Aguilar J Springer TA . Genomic structure of an integrin alpha subunit, the leukocyte p150,95 molecule. J Biol Chem (1990) 265:2782–8. doi: 10.1016/S0021-9258(19)39870-9 2303426

[19] Corbi AL Miller LJ O'Connor K Larson RS Springer TA . cDNA cloning and complete primary structure of the alpha subunit of a leukocyte adhesion glycoprotein, p150,95. EMBO J (1987) 6:4023–8. doi: 10.1002/j.1460-2075.1987.tb02746.x PMC553883 3327687

[20] Hou L Voit RA Sankaran VG Springer TA Yuki K . CD11c regulates hematopoietic stem and progenitor cells under stress. Blood Adv (2020) 4:6086–97. doi: 10.1182/bloodadvances.2020002504 PMC7757003 33351105

[21] Nakayama M . Antigen presentation by MHC-dressed cells. Front Immunol (2015) 5:672. doi: 10.3389/fimmu.2014.00672 25601867 PMC4283639

[22] Ueno T Saito F Gray DH Kuse S Hieshima K Nakano H . CCR7 signals are essential for cortex-medulla migration of developing thymocytes. J Exp Med (2004) 200:493–505. doi: 10.1084/jem.20040643 15302902 PMC2211934

[23] Nitta T Nitta S Lei Y Lipp M Takahama Y . CCR7-mediated migration of developing thymocytes to the medulla is essential for negative selection to tissue-restricted antigens. Proc Natl Acad Sci U S A (2009) 106:17129–33. doi: 10.1073/pnas.0906956106 PMC2761327 19805112

[24] Kappler JW Roehm N Marrack P . T Cell tolerance by clonal elimination in the thymus. Cell (1987) 49:273–80. doi: 10.1016/0092-8674(87)90568-X 3494522

[25] Liston A Lesage S Wilson J Peltonen L Goodnow CC . Aire regulates negative selection of organ-specific T cells. Nat Immunol (2003) 4:350–4. doi: 10.1038/ni906 12612579

[26] Santamaria JC Borelli A Irla M . Regulatory T cell heterogeneity in the thymus: Impact on their functional activities. Front Immunol (2021) 12:643153. doi: 10.3389/fimmu.2021.643153 33643324 PMC7904894

[27] Jordan MS Boesteanu A Reed AJ Petrone AL Holenbeck AE Lerman MA . Thymic selection of CD4+CD25+ regulatory T cells induced by an agonist self-peptide. Nat Immunol (2001) 2:301–6. doi: 10.1038/86302 11276200

[28] Wang H Zuniga-Pflucker JC . Thymic microenvironment: Interactions between innate immune cells and developing thymocytes. Front Immunol (2022) 13:885280. doi: 10.3389/fimmu.2022.885280 35464404 PMC9024034

[29] Hernandez JB Newton RH Walsh CM . Life and death in the thymus–cell death signaling during T cell development. Curr Opin Cell Biol (2010) 22:865–71. doi: 10.1016/j.ceb.2010.08.003 PMC2993787 20810263

[30] Eisenbarth SC . Dendritic cell subsets in T cell programming: location dictates function. Nat Rev Immunol (2019) 19:89–103. doi: 10.1038/s41577-018-0088-1 30464294 PMC7755085

[31] Oh J Shin JS . The role of dendritic cells in central tolerance. Immune Netw (2015) 15:111–20. doi: 10.4110/in.2015.15.3.111 PMC4486773 26140042

[32] Yasutomo K Lucas B Germain RN . TCR signaling for initiation and completion of thymocyte positive selection has distinct requirements for ligand quality and presenting cell type. J Immunol (2000) 165:3015–22. doi: 10.4049/jimmunol.165.6.3015 10975810

[33] Ladi E Schwickert TA Chtanova T Chen Y Herzmark P Yin X . Thymocyte-dendritic cell interactions near sources of CCR7 ligands in the thymic cortex. J Immunol (2008) 181:7014–23. doi: 10.4049/jimmunol.181.10.7014 18981121

[34] Xing Y Wang X Jameson SC Hogquist KA . Late stages of T cell maturation in the thymus involve NF-kappaB and tonic type I interferon signaling. Nat Immunol (2016) 17:565–73. doi: 10.1038/ni.3419 PMC4837029 27043411

[35] Hou L Rao DA Yuki K Cooley J Henderson LA Jonsson AH . SerpinB1 controls encephalitogenic T helper cells in neuroinflammation. Proc Natl Acad Sci U S A (2019) 116:20635–43. doi: 10.1073/pnas.1905762116 PMC6789640 31548399

