# Supplementary material for: CD11c regulates late-stage T cell development in the thymus
Source: Front Immunol. 2022 Nov 10;13:1040818. doi: 10.3389/fimmu.2022.1040818 (PMC9684328; doi:10.3389/fimmu.2022.1040818)

CD11c regulates late-stage T cell development in the thymus

Author: Lifei Hou* and Koichi Yuki*

Affiliation: Department of Anesthesiology, Critical Care and Pain Medicine, Cardiac Anesthesia Division, Boston Children’s Hospital; Departments of Anaesthesia and Immunology, Harvard Medical School

Corresponding author: L.H. ([Lifei.hou@childrens.harvard.edu](mailto:Lifei.hou@childrens.harvard.edu))

K.Y ([Koichi.Yuki@childrens.harvard.edu)](mailto:Koichi.Yuki@childrens.harvard.edu))

Running title: CD11c maintains T cell survival

Supplement Figure 1. Gating strategy for identifying thymic DC subsets.


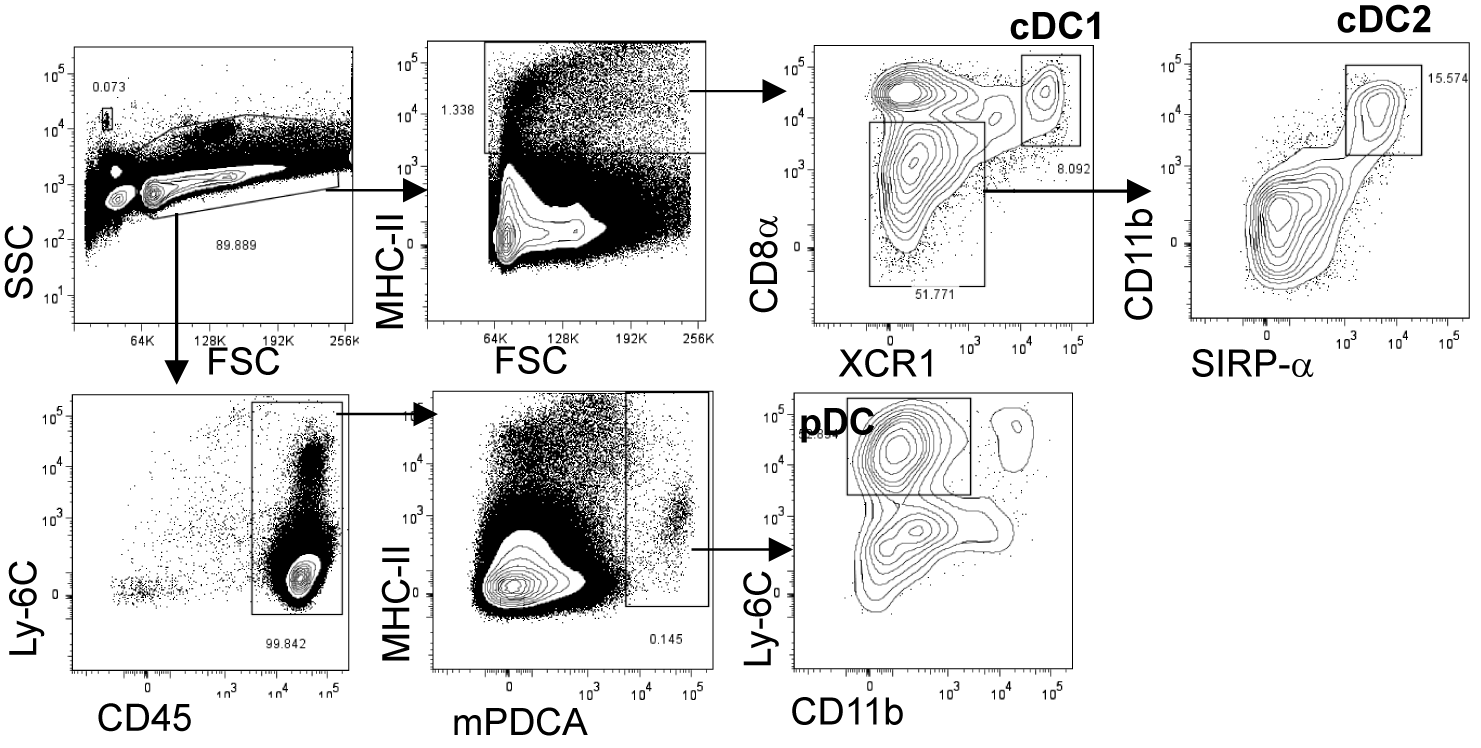

Supplement: Supplementary file 1 [file DataSheet_1.docx]
